# Real-World Analysis of Survival and Clinical Events in a Cohort of Italian Perinatally HIV-1 Infected Children From 2001 to 2018

**DOI:** 10.3389/fped.2021.665764

**Published:** 2021-07-16

**Authors:** Elena Chiappini, Francesca Larotonda, Catiuscia Lisi, Vania Giacomet, Paola Erba, Stefania Bernardi, Paola Zangari, Antonio Di Biagio, Lucia Taramasso, Carlo Giaquinto, Osvalda Rampon, Clara Gabiano, Silvia Garazzino, Claudia Tagliabue, Susanna Esposito, Eugenia Bruzzese, Raffaele Badolato, Domenico Zanaboni, Monica Cellini, Maurizio Dedoni, Antonio Mazza, Andrea Pession, Anna Maria Giannini, Filippo Salvini, Icilio Dodi, Ines Carloni, Salvatore Cazzato, Pier Angelo Tovo, Maurizio de Martino, Luisa Galli

**Affiliations:** ^1^Paediatric Infectious Diseases Unit, Department of Health Sciences, Anna Meyer Children's Hospital, University of Florence, Florence, Italy; ^2^Department of Paediatric Medicine, Anna Meyer Children's Hospital, University of Florence, Florence, Italy; ^3^Paediatric Infectious Diseases Unit, Department of Paediatrics, Luigi Sacco Hospital, University of Milan, Milan, Italy; ^4^Unit of Immune and Infectious Diseases, Stefania Bernardi Academic Department of Pediatrics (DPUO), Bambino Gesù Children's Hospital, Istituti di Ricovero e Cura a Carattere Scientifico, Rome, Italy; ^5^Research Unit of Clinical Immunology and Vaccinology, Paola Zangari Academic Department of Pediatrics (DPUO), Bambino Gesù Children's Hospital, Istituti di Ricovero e Cura a Carattere Scientifico, Rome, Italy; ^6^Infectious Diseases Unit, Policlinico San Martino Hospital, University of Genova, Genoa, Italy; ^7^Department of Women and Child Health, University of Padova, Padua, Italy; ^8^Paediatric Infectious Diseases Unit, Regina Margherita Children's Hospital, University of Turin, Turin, Italy; ^9^Paediatric Highly Intensive Care Unit, Department of Pathophysiology and Transplantation, Istituti di Ricovero e Cura a Carattere Scientifico Ca' Granda Ospedale Maggiore Policlinico Foundation, University of Milan, Milan, Italy; ^10^Paediatric Department, Pietro Barilla Children's Hospital, University of Parma, Parma, Italy; ^11^Paediatric Unit, Department of Translational Medical Sciences, University of Naples Federico II, Naples, Italy; ^12^Department of Clinical and Experimental Sciences, University of Brescia, Brescia, Italy; ^13^Department on Internal Medicine and Therapeutics, Istituti di Ricovero e Cura a Carattere Scientifico Policlinico “S. Matteo” Foundation, University of Pavia, Pavia, Italy; ^14^Paediatric Hemato-Oncology Unit, Department of Medical and Surgical Sciences, University of Modena and Reggio Emilia, Modena, Italy; ^15^Department of Paediatrics, Ospedale Microcitemico, Cagliari, Italy; ^16^Department of Paediatrics, “S. Chiara” Hospital, Trento, Italy; ^17^Paediatric Unit, IRCCS Scientific Institute for Research and Healthcare, Sant'Orsola Hospital, Bologna, Italy; ^18^Paediatric Infectious Diseases Unit, University Hospital Policlinico Giovanni XXIII, Bari, Italy; ^19^Department of Paediatrics, Niguarda Hospital, University of Milan, Milan, Italy; ^20^Department of Medicine and Surgery, Pietro Barilla Children's Hospital, University of Parma, Parma, Italy; ^21^Department of Mother and Child Health, Salesi Children's Hospital, Ancona, Italy

**Keywords:** HIV, perinatal infection, children, antiretroviral therapy (ART), aids, survival, epidemiology

## Abstract

**Background:** Combined antiretroviral therapy (cART) has been associated with a steep decrease in mortality and morbidity in HIV-1 infected children. New antiretroviral molecules and drug classes have been developed and the management of HIV-infected children has improved, but recent data on survival are limited.

**Methods:** An observational retrospective study investigating changes in mortality and morbidity was conducted on 1,091 perinatally HIV-1 infected children enrolled in the Italian Register for HIV Infection in Children and followed-up from 2001 to 2018.

**Results:** Three hundred and fifty-four (32%) AIDS events and 26 (2%) deaths occurred overtime. Mortality rates decreased from 0.4/100 person-years in 2001–2006 to 0.27/100 person-years in 2007–2012 and 0.07/100 person-years in 2013–2018. Notably, 92% of the dead children were born in Italy, but only 50% were followed-up since birth or within three months of age. Seventy three percent of children had started cART at age ≥6 months; 23% were treated for <30 days before death. B and C clinical events progressively decreased (*P* < 0.0001). Opportunistic infections significantly decreased over time, but still were the most common events in all the periods (6.76/100 person-years in 2013–2018). In the last period, severe bacterial infections were the most common ones. Cancer rates were 0.07/100; 0.17/100; 0.07/100 person-years in the three periods, respectively.

**Conclusions:** Progressive reductions both in mortality and in rates of class B and C clinical events and OIs have been observed during the cART era. However, deaths were still registered; more than half of dead children were enrolled after birth and had belatedly started cART.

## Introduction

The cART was introduced in 1996 and, thanks to the use of different agents targeting various steps of the HIV life cycle, had a material impact on the HIV infection pandemic (1). From 1996 to nowadays, numerous aspects regarding the treatment of the HIV infected paediatric population changed. New antiretroviral drug classes have been introduced (such as integrase inhibitors) (2, 3); the proportion of children receiving cART increased and the time of exposure to cART is extending (4, 5); several paediatric 2-in-1 and 3-in-1 fixed-dose combinations (FDC) have become available and have received quality certification by the WHO and FDA for use in children; the development of paediatric antiretroviral drugs and formulations has been prioritised by WHO, which published a paediatric drug development toolkit summarising the challenges and solutions for promoting and accelerating development of antiretroviral formulations suitable for infants, children, and adolescents (6, 7). Mortality rates and rates of hospitalisation and progression to AIDS dramatically decreased from the pre-cART era to the cART era, in parallel with better viral suppression and higher CD4^+^ T-cells counts (8–10). In particular, the prognosis of HIV-1 infected children has considerably improved thanks to early treatment with cART (11). It has been demonstrated that early HIV diagnosis and early initiation of antiretroviral therapy reduce infant mortality by 76% and HIV progression by 75% (12). The benefit of treating HIV-infected infants early in life has been quantified in a large population of 210 infants from 11 European countries: the risk of developing AIDS or death at 1 year was 1.6% in infants treated before 3 months of life and 11.7% in infants treated later (*P* < 0.001) (11). Initiating cART as soon as possible after birth (in particular before the age of 5 months) has also been related to a better recovery of CD4+ cell count (13, 14).

However, non-AIDS related deaths (associated to metabolic complications, drug induced adverse events or organ failures) are emerging and their number is increasing (15–17). From 1996 onward, incidence rates of opportunistic infections (OIs) decreased, especially central nervous system infections, candidiasis, lymphoid interstitial pneumonia, *Pneumocystis jirovecii* pneumonia, and disseminated TB (18–21). Incidence rates of HIV associated nephropathy (HIVAN) and HIV encephalopathy (HIVE) significantly decreased, too (22–25). However, new aspects are emerging: in the low-to-middle income countries (LMICs) the incidence of bacterial pneumonia, tuberculosis, candidiasis and varicella remained stable over years despite the use of cART (26) and chronic lung disease is emerging among older children and adolescents (27); other types of renal disease (i.e., tubular dysfunction, human immunodeficiency virus immune complex kidney disease, urinary tract infections) (28–30) and milder neurocognitive deficits (i.e., lower total intelligence quotient, language impairment, poorer working memory) (31–37) are occurring always more frequently. Cancer rates considerably decreased after year 2000, when the cART use was widely diffused (38–40). The incidence rates of AIDS-defining cancers have been deeply reduced during the cART era but non-AIDS-defining cancers (such as Hodgkin lymphoma, leiomyosarcoma and other sarcomas, hepatocarcinoma) surprisingly increased (41–43). Incidence rates of malignancies in HIV-infected children are, still today, much higher than those in the healthy paediatric population (44).

Monitoring the variations of HIV associated symptoms and signs, collecting relevant clinical events and laboratory information, understanding the new aspects of the disease is extremely important to investigate the impact of cART on the HIV infected paediatric population and to improve children's quality of life and life expectancy. An observational population study was previously conducted (19) on perinatally HIV-1 infected children enrolled in *the Italian Register for HIV Infection in Children* from 1985 to 2005, analysing survival rates and CDC class B and C clinical events during the pre-cART and cART eras. Progressive reduction in mortality rates and clinical events was reported, while the occurrence of OIs (particularly severe bacterial infections and pneumonia) still was at higher rates. The purpose of this study is to continue investigating how clinical events among HIV-infected children are changing in the most recent years.

## Materials and Methods

### Study Design

An observational population study was conducted on perinatally HIV-1 infected children enrolled in *the Italian Register for HIV Infection in Children* from 2001 to 2018. Data from children prospectively followed from birth were analysed separately. Children lost to follow-up were censored at last cheque. The rates of CDC class B and C clinical events, including malignancies, organ-specific complications and OIs, were analysed. Incidence rates of deaths and clinical events were compared to data collected from 1985 to 2005 in the *Italian Register for HIV Infection in Children* and published on AIDS Journal in 2007 (19).

### Data Collection

Data were collected by *the Italian Register for HIV Infection in Children*, which is a nationwide multicentre study of children exposed to HIV-1 instituted in 1985 by the Italian Society of Paediatrics. The data source is a network of 106 paediatric clinics distributed throughout Italy, and it has been proved to be highly representative (86.5%) of the entire Italian population of HIV-1-infected children (9). The data are transmitted to the two coordinating centres, the Departments of Paediatrics in the Universities of Florence and Turin. In this study data collected from all perinatally infected children never lost to follow-up up to December 31, 2018 were analysed.

### Laboratory Investigations

CD4^+^ T-lymphocytes and HIV-RNA viral loads (VL) were measured by each Italian centre involved in the study with standardised methods. According to the US guidelines for the use of antiretroviral agents in paediatric HIV-1 infection, CD4^+^ T-cell percentages, rather than their absolute counts, were utilised, as these percentages reflect the immune status of HIV-1-infected children more accurately.

### Treatment

The specific therapy offered was based upon decision criteria proposed by the Italian and US guidelines (15, 16). The cART was defined as a combined antiretroviral therapy consisting of at least three antiretroviral drugs of two different classes (e.g., protease inhibitors, nucleoside reverse transcriptase inhibitors, non-nucleoside reverse transcriptase inhibitors, and integrase inhibitors). Moreover, several two-drugs based regimens were considered as cART (i.e., darunavir/cobicistat or darunavir/ritonavir-raltegravir; darunavir/cobicistat or darunavir/ritonavir-dolutegravir; lamivudine-darunavir/cobicistat; lamivudine-darunavir/ritonavir). Dual therapy regimens with lamivudine and dolutegravir have been included.

### Calendar Periods

Clinical event rates were analysed starting from 2001 to 2018. This time was subdivided into three periods: from 2001 to 2006, from 2007 to 2012 and from 2013 to 2018, each one six-year-long.

### Definitions

Clinical events were diagnosed, at each participating centre, by a paediatrician and infectious diseases specialist, expert in HIV-1 infection. The diagnoses were based on the 1994 CDC classification and definitions (17), as follows: AIDS was defined as the first-class C event according to the CDC's definition; class B, and C clinical events and HIV-1-related organ-specific complications (i.e., lymphoid interstitial pneumonia, HIV-1-related encephalopathy, cardiomyopathy, hepatopathy, nephropathy) were strictly defined according to the recommendations laid down by the CDC. All the clinical events that did not fit CDC definitions, were reported as “non-AIDS related” events. Haematological alterations (HIV-1-related anaemia, granulocytopenia, and thrombocytopenia) were evaluated in total. Malignancies included all cancers developed during the observation time. Both AIDS-defining cancers (brain lymphomas, small non-cleaved cell non-Hodgkin lymphoma, immunoblastic or large-cell lymphoma of B cell or unknown immunological phenotype and Kaposi sarcoma) and other cancers (i.e., leiomyosarcoma, cervical carcinoma, hepatoblastoma, or acute lymphoblastic leukaemia) were considered. OIs included candidiasis (considering separately as persistent/recurrent oral or skin candidiasis and oesophageal/tracheobronchial disease), cytomegalovirus-related disease (cytomegalovirus retinitis and all other forms of cytomegalovirus disease other than retinitis, excluding congenital cytomegalovirus infection), cryptosporidiosis, polydermatomeric herpes zoster, disseminated herpes simplex, *Pneumocystis jirovecii* pneumonia, systemic fungal infection (cryptococcosis, coccidioidomycosis, histoplasmosis), progressive multifocal leukoencephalopathy, disseminated *Mycobacterium avium complex*, and serious bacterial infections. The last included bacteraemia, internal organ abscess, meningitis, osteomyelitis, ascertained or presumptive bacterial pneumonia, septic arthritis, pyelonephritis, and mastoiditis.

### Statistical Analysis

Age, VL, and CD4^+^ T-lymphocyte percentage were expressed as the median and interquartile range (IQR). VL data were provided by the centres belonging to *the Italian Register for HIV Infection in Children*. The cut-off for undetectable VL was different in the various laboratories and varied over time. In particular, VL was defined undetectable when <400 copies/mL in 3% of undetectable cases, VL <80 copies/mL in 0.15%, VL <50 copies/mL in 51%, VL <40 copies/mL in 17%, VL <37 copies/mL in 10%, VL <34 copies/mL in 5%, VL <20 copies/mL in 12%, VL <3 copies/mL in 2%. Viremia was assessed considering the most recent viremia within 6 months. Clinical events were assigned to the period during which they occurred and the number of events per 100 person-years of observation time in the corresponding period was calculated. Event rates were reported as rates per 100 person-years and 95% confidence intervals (CI) were calculated. Additionally, the incidence rate ratio (IRR) was calculated. A single patient could contribute to multiple events (except for the mortality analysis) to the same or different periods; consequently, a Poisson regression model, adjusted for age and correlated observation across time within the same patient, was used to compare morbidity rates among the study periods. Therefore, the adjusted rate ratio (RR) was calculated. To test the influence of co-variables and to identify factors associated with OIs or cancer, a multivariate analysis was performed using the Poisson regression model, as indicated by the dichotomous nature of the dependent variable. For insertion into the model, only variables with a critical value of *P* < 0.05 were considered. Statistical analyses were performed using STATA/SE, version 13.0 (Stata Corporation, College Station, Texas, USA). *P* < 0.05 was considered statistically significant.

## Results

### Study Population

**Figure 1:**
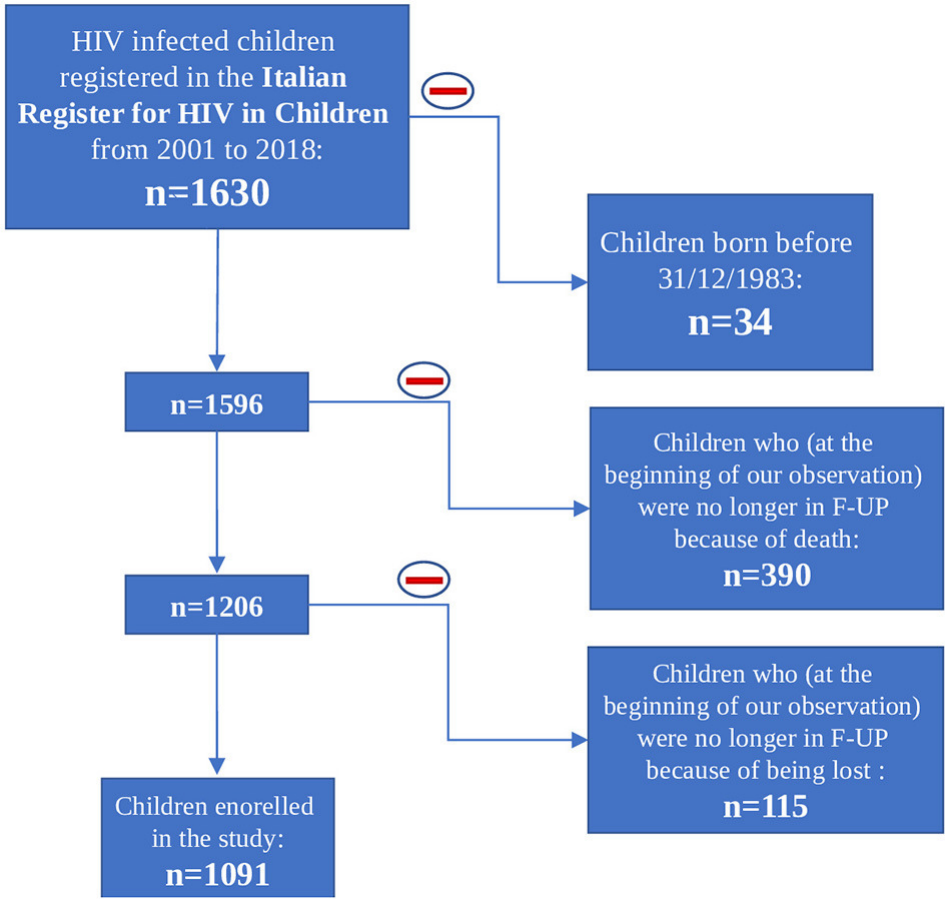
Children included and excluded from the study population.

**Figure 2:**
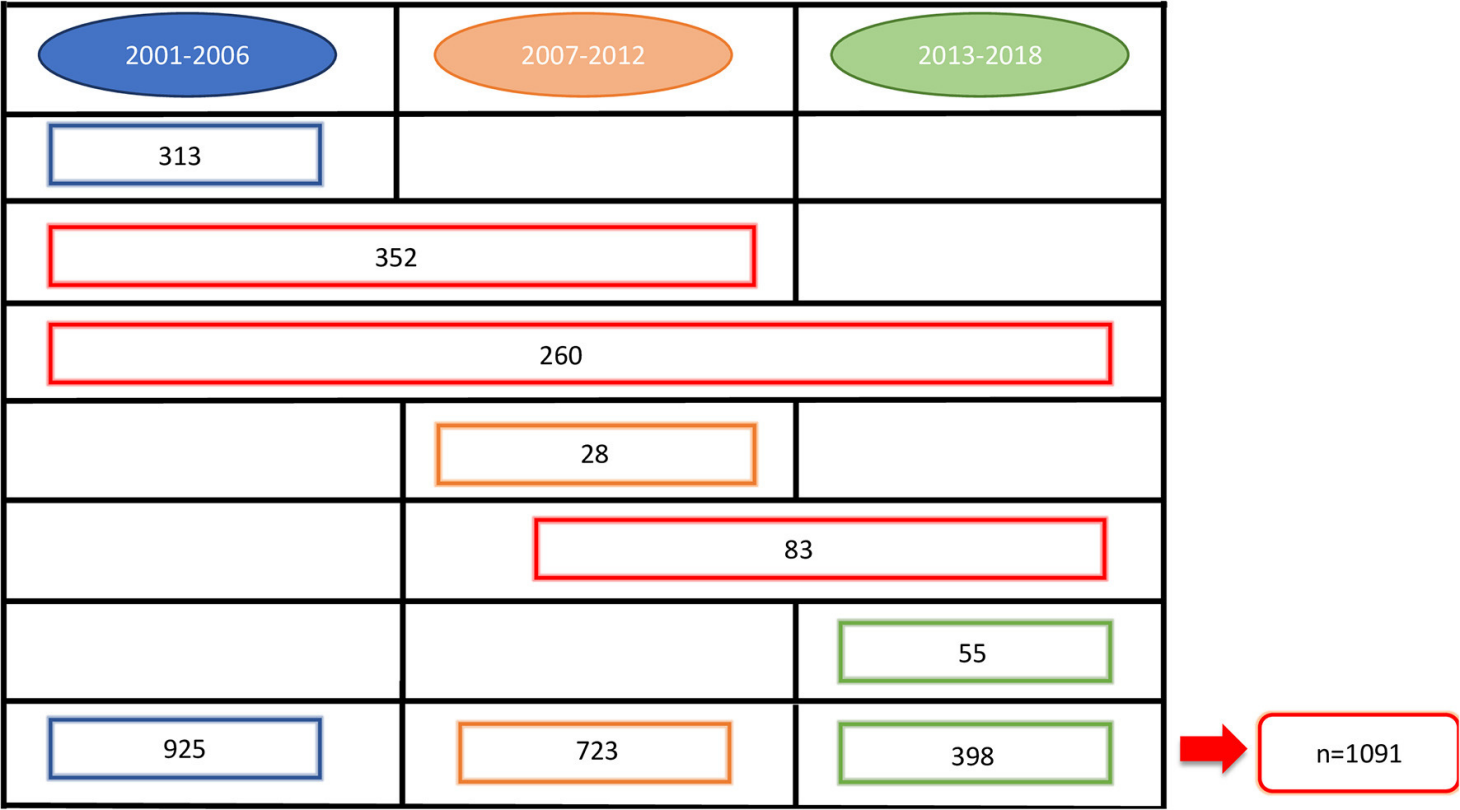
Number of children who contributed to the three periods of analysis.

At the beginning of our observation, in 2001, 1,630 perinatally HIV-1 infected children were enrolled in the Italian Register for HIV Infection in Children. We excluded from our study population all the children born before 31/12/1983 (*n* = 34) because they already were over 18 years old. Moreover, 505 children were excluded because no longer in follow up from 2001 onwards: 390 because of death and 115 because lost to follow up. The remaining 1,091 HIV-1 infected children were enrolled in our study. Among these 1,091 children, 276 (25.3%) were lost to follow up, 77 (7.1%) of whom were followed up from birth (Figure 1). Nine hundred thirty-five children were part of the first study period, 723 of the second one and 398 of the third one. Three hundred ninety-six children contributed to only one period of analysis, 435 children to two periods and 260 children to three periods (Figure 2). Three hundred forty-three (37.1%) children belonging to the first study period were followed up from birth, 281 (38.7%) children were in follow up from birth during the second period and 133 (33.4%) during the third one (Table 1). Seven hundred twenty-seven children were already in the Italian Register for HIV Infection in Children before 2001. New enrolments during the whole study period (2001–2018) were 396: 313 in the first period, 28 in the second one and 55 in the third one (Table 1). All the data regarding children over 18 years old were excluded from the analysis, in order to specifically characterise paediatric population. Each child contributed to the study for 7.5 years (median; IQR: 3.7–11.2) and total observation time was 8480.4 years. Overall, 26 (2.4%) children died during the study period. The characteristics of our study population are summarised in Table 1.

**Table 1:** Characteristics of the 1,091 HIV-1 infected children, by calendar period of observation.

	**2001–2006**	**2007–2012**	**2013–2018**	**All children**
	***n* (95 % CI)**	***n* (%)**	***n* (%)**	***n* (%)**
No. of children	925	723	398	1,091
Observation time (person-years)	4176.1	2899.0	1405.4	8480.4
Followed-up from birth [*n* (%)]	343 (37.1)	281 (38.7)	133 (33.4)	757 (69.4)
Lost to follow-up during the study period [*n* (%)]	145 (15.7)	89 (12.3)	42 (10.6)	276 (25.3)
New enrolled [*n* (%)]	313 (33.8)	28 (3.9)	55 (13.8)	396 (36.3)
Median CD4^+^ T-lymphocytes [% (IQR)^*^]	28 (21–36)	32 (26–38)	34 (29–39.6)	31 (24–37)
Median CD4^+^ T-lymphocytes at last cheque [% (IQR)^*^]	29 (22–36)	33 (27–39)	34.3 (29.2–40.8)	31 (24–38)
Median viral load [copies/mL (IQR)^*^]	872 (49–15.000)	49 (39–399)	48 (33–49)	150 (49–4.630)
Median viral load [Log (IQR)^*^]	2.94 (1.69–4.18)	1.69 (1.59–2.60)	1.68 (1.52–1.69)	2.18 (1.69–3.67)
Median viral load at last cheque [Log (IQR)^*^]	2.31 (1.69–3.93)	1.69 (1.59–2.47)	1.59 (1.52–1.69)	1.69 (1.59–3.02)
Viral load at last cheque detectable	467	230	63	403 (36.9)
Undetectable	428	477	260	681 (62.4)
Deaths [*n* (%)]	17 (1.84)	8 (1.11)	1 (0.25)	26 (2.38)
Median age at death (<18 years) [years (IQR)^*^]	12.1 (1.3–16.5)	14.3 (6.7–15.3)	3.5(3.5–3.5)	12.9 (1.3–15.6)
Receiving HAART in the study period [*n* (%)]	788 (85.2)	594 (82.2)	372 (93.5)	1,023 (93.8)
Median age at starting HAART [years (%)]	7.2 (3.2–10.7)	4.8 (1.8–8.2)	2.2 (0.5–5.6)	6.5 (2.3–10.4)
Duration of HAART (person years)	3174.7	2491.6	1528.2	7,194

* *IQR, interquartile range*.

### Analysis of Survival

Among the 1,091 children, 925 patients were observed in the period 2001–2006; 723 in 2007–2012; and 398 in 2013–2018. Within the same periods, 17, 8, and 1 deaths occurred, respectively, with a significantly decreased rate of death overtime. Age adjusted mortality rates decreased from 0.4/100 person-years (CI: 0.24–0.64) in 2001–2006 period to 0.27/100 person-years (CI: 0.13–0.52) in 2007–2012 and to 0.07/100 person-years (CI: 0.036–3.5) in 2013–2018 (Table 2). Poisson regression model adjusted for sex, age at the first medical examination, place of birth, use of cART, viral load, and CD4^+^ T-lymphocytes percentage was used to compare death rates among the study periods. Significantly reduced rates of death were observed in children with high CD4^+^ T-lymphocyte percentage (>15%) (Table 2). Differences in mortality risk between patients born before and after 1996 (year of cART introduction) were analysed, however there was no significant decrease in incidence rate ratio between these two cohorts (Table 2). Mortality risk in children not in follow up from birth was higher, even if not significantly, than that in children followed up from birth (mortality IRR of children followed up after birth: 1.36; *P* value 0.536) (Table 2). Reported causes of mortality were opportunistic infections (*n* = 8), specific or multi-organ failures (*n* = 5), cancer (*n* = 3), sepsis (*n* = 3), wasting syndrome (*n* = 2), cardiomyopathy and cardiac disease (*n* = 2), acute pulmonary oedema (*n* = 2), massive haemorrhages (*n* = 1), consequences of encephalopathy (*n* = 1), lactic acidosis (*n* = 1), lymphoid interstitial pneumonia (*n* = 1), not known (*n* = 3). Fifteen out of 26 dead children (58%) died when they were >10 years old while 6 out of 26 (23%) died when they were ≤ 1-year-old. Only eight (30.8%) of the 26 dead children were followed up from birth. Nineteen (73%) had initiated cART at age ≥6 months. 6 children (23%) were treated for <30 days before death, 12 (46%) for ≥10 years and 7 (27%) for >1 year but <10 years. Almost all the children (*n* = 23/26; 88%) presented detectable VL at the last cheque before death and 15/26 children (58%) had CD4^+^T-cell counts <15% at last cheque. Only three children had undetectable VL at the last cheque before death. However, in these 3 patients, VL reported 12–18 months earlier were detectable (Table 3).

**Table 2:** Mortality rates among the study periods adjusted for age.

**Factors**	**Events (*n*)**	**Person-years**	Univariate IRR^*^ (95%CI^*^)	***P***	Multivariate IRR^*^ (95%CI^*^)	***P***
**Period**						
2001–2006	17	4176.1	1		1	
2007–2012	8	2899.0	0.71 (0.26–1.89)	0.489	0.97 (0.23–4.10)	0.971
2013–2018	1	1405.4	0.06 (0.01–0.43)	0.006	0.07 (0.01–0.64)	0.019
**Sex**						
Male	12	4021.5	1			
Female	14	4458.9	1.30 (0.51–3.32)	0.577		
**Follow-up**						
From birth	8	3365.4	1			
After birth	18	5115.0	1.36 (0.51–3.65)	0.536		
**Place of birth**						
Italy	24	7026.4	1			
Abroad	2	1454.0	0.37 (0.08–1.80)	0.220		
**Viral load**						
Undetectable	2	3043.3	1		1	
Detectable	15	1854.4	6.45 (1.38–30.11)	0.018	2.08 (0.40–10.95)	0.387
Missing	9	3582.7	2.54 (0.54–12.01)	0.241	1.22 (0.23–6.51)	0.818
**CD4**^**+**^ **T-lymphocyte percentage at the last cheque**						
15%	13	336.0	1		1	
>15%	4	4434.8	0.02 (0.003–0.08)	<0.0001	0.03 (0.01–0.11)	<0.0001
Missing	9	3709.6	0.08 (0.03–0.22)	<0.0001	0.19 (0.03–1.32)	0.093
**Date of birth**						
After 1996	16	4611.8	1			
Before 1996	10	3868.6	1.31 (0.52–3.32)	0.565		

* *IRR, incidence rate ratio; CI, confidence interval*.

**Table 3:** Characteristics of deaths in perinatally HIV-1 infected children (from 2001 to 2018).

	**1**	**2**	**3**	**4**	**5**	**6**	**7**
Sex	F	M	M	M	F	M	M
Child's/Mother's origin	Italy/Italy	Abroad/Abroad	Italy/Abroad	Italy/Italy	Italy/Italy	Abroad/Abroad	Italy/Italy
Clinical and immun.^*^ staging	C 3	B 1	C 3	C 3	C 3	B 3	C 3
Start of observation	After birth	After birth	After birth	From birth	After birth	After birth	From birth
First observation	1986	1999	2002	1989	1991	1992	2004
Age at first observation	2 months	3 years	3 months	0 months	5 years	13 months	1 month
Start of treatment	1990	1999	2002	1991	1991	1997	2004
Age start of treatment	4 years	3 years	3 months	19 months	6 years	6 years	6 months
End of treatment	2001	2001	2002	2002	2002	2003	2004
Last therapy	DDI+ D4T+ NFV	AZT+ 3TC+ DMP	AZT+ 3TC+ NFV	AZT+ 3TC+ SQV	DDI+ D4T+ TDF+ APV+ RTV	3TC+ D4T+ NFV	DDI+ 3TC+ NFV
Death	2001	2001	2002	2002	2002	2003	2004
Age at death	15 years	5 years	3 months	13 years	17 years	12 years	7 months
T-lymph.^*^ CD4^+^ percentage	2%	33%	12%	11%	2%	2%	6%
Viral load (detect.^*^/undet.^*^)	Detect.,	Detect.,	Detect.,	Detect.,	Detect.,	Detect.,	Detect.,
Cause of death	Unspecified	Cardiomiopathy	*Pneumocystis jirovecii* pneumonia	Hepatic failure	Lactic acidosis	Heart disease Pulmonary edoema	Bacterial pneumonia
	**8**	**9**	**10**	**11**	**12**	**13**	**14**
Sex	F	M	F	M	F	F	F
Child's/Mother's origin	Italy/Abroad	Italy/Italy	Italy/Italy	Italy/Italy	Italy/Italy	Italy/Italy	Italy/Italy
Clinical and immun.^*^ staging	C 3	C 3	C 3	C 3	C 3	C 3	C 2
Start of observation	After birth	After birth	From birth	After birth	From birth	From birth	From birth
First observation	2004	2004	1996	1989	1993	2005	1989
Age at first observation	4 months	5 months	0 months	12 months	0 months	0 months	0 months
Start of treatment	2004	2004	1996	1990	1993	2005	2000
Age at start of treatment	4 months	5 months	7 months	1 year	3 months	3 months	11 years
End of treatment	2004	2004	2004	2004	2005	2006	2006
Last therapy	AZT+ 3TC+ LPV+ RTV	AZT+ 3TC+ NFV	AZT+ 3TC+ LPV+ RTV	3TC+ RTV+ TPV	ABC+3TC+ LPV+ RTV	AZT+ 3TC+ NFV	FTC+ DMP+ TDF
Death	2004	2004	2004	2004	2006	2006	2006
Age at death	4 months	5 months	8 years	16 years	13 years	15 months	17 years
T-lymphocyte CD4^+^ percentage	5%	8%	3%	3%	16%	28%	12%
Viral load (detect.^*^/undet.^*^)	Detect.	Detect.	Detect.	Undet.	Detect.	Detect.	Detect.
Cause of death	*Pneumocystis jirovecii* pneumonia	*Pneumocystis jirovecii* pneumonia	Acute pulmonary edoema	Acute renal failure TBC (typic and atypic) Wasting syndrome	Invasive candidiasis	Hepatic failure Sepsis	Unspecified
	**15**	**16**	**17**	**18**	**19**	**20**	**21**
Sex	F	M	M	F	M	M	F
Child's/Mother's origin	Italy/Italy	Italy/Italy	Italy/Italy	Italy/Italy	Italy/Italy	Italy/Italy	Italy/Italy
Clinical and immun.^*^ staging	C 3	C 3	C 2	B 3	C 2	C 3	C 2
Start of observation	After birth	After birth	After birth	After birth	From birth	After birth	After birth
First observation	1992	1990	1999	2008	1994	1997	2009
Age at first observation	3 years	11 months	5 months	3 months	0 months	10 months	3 months
Start of treatment	1993	1993	2000	2008	1995	1997	
Age at start of treatment	3 years	3 years	11 months	4 months	9 months	12 months	
End of treatment	2006	2006	2006	2008	2009	2009	
Last therapy	AZT+ 3TC+ APV+ RTV	3TC+ NVP+ APV	AZT+ DDI+ NFV	3TC+ NVP	FTC+ TDF+ LPV+ RTV	3TC+ TDF+ DRV+ RTV	
Death	2006	2006	2006	2008	2009	2009	2009
Age at death	17 years	16 years	7 years	4 months	15 years	12 years	4 months
T-lymphocyte CD4^+^ percentage	3%	20%	48%	6%	35%	3%	20%
Viral load (detect.^*^/undet.^*^)	Detect.	Detect.	Undet.	Detect.	Undet.	Detect.	Detect.
Cause of death	Wasting syndrome	Lymphocytic interstitial pneumonia	Bacterial pneumonia	Massive hemorrages	Burkitt lymphoma	Sepsis	Disseminated *Cytomegalovirus* Heart, pulmonary, circulatory failure
	**22**	**23**	**24**	**25**	**26**	–	–
Sex	F	F	F	F	M	–	
Child's/Mother's origin	Italy/Italy	Italy/Italy	Italy/Italy	Italy/Italy	Italy/Abroad	–	–
Clinical and immunological staging	C 2	C 3	A 2	C 3	C 1	–	–
Start of observation	From birth	After birth	After birth	After birth	After birth	–	–
First observation	1998	1996	2002	2010	2010	–	–
Age at first observation	0 months	2 months	6 years	13 years	4 months	–	–
Start of treatment	1998	1997	2002	2010	2011	–	–
Age at start of treatment	10 months	9 months	6 years	13 years	7 months	–	–
End of treatment	2011	2010	2012	2012	2013	–	–
Last therapy	AZT+ ABC+ 3TC	DDI+ 3TC+ LPV+ RTV	DDI+ D4T+ LPV+ RTV	DDI+ 3TC+ ATV+ MK0	AZT+ 3TC+ LPV+ RTV	–	–
Death	2011	2012	2012	2012	2013	–	–
Age at death	13 years	16 years	15 years	14 years	3 years	–	–
T-lymphocyte CD4^+^ percentage	33%	18%	31%	5%	47%	–	–
Viral load (detect.^*^/undet.^*^)	Detect.	Detect.	Detect.	Detect.	Detect.	–	–
Cause of death	Heart, pulmonary and circulatory failure	Consequences of encephalopathy	Non-Hodgkin B-cell lymphoma	Kaposi sarcoma	Unspecified	–	–

* *Immun., immunological; lymph, lymphocyte; detect., detectable; undet., undetectable*.

### Modifications of Clinical Event Rates

Progressive significant reductions in CDC class B and C clinical event rates were observed during the study periods (Table 4). One hundred eighty class B events occurred during the first period (4.31/100 person-years), while only 25 were observed in 2013–2018 (1.78/100 person-years), demonstrating a significant decrease over time (*P* < 0.0001). Class C events were 229 in the first period (5.48/100 person-years) and considerably decreased (*P* < 0.0001) to 42 in the last one (2.99/100 person-years). Poisson regression model adjusted for timing of follow up and date of birth (before or after 1996) was used to compare class B and C event rates among the study periods. We noticed that the IRR for both B and C clinical events was significantly lower in children followed up from birth, as well as being progressively lower in more recent calendar periods. On the other hand, children born after 1996 didn't have significantly decreased incidence rates of class B and C events (Table 5).

**Table 4:** Centres for disease control and prevention class B and C clinical events in HIV-1 infected children by calendar period.

**Event class and calendar period**	**Events (*n*)**	**Person-years**	**Rate ratio (events/100 person-years)**	Adjusted^a^ RR^*^ (95% CI^*^)	***P***
**Class B**					
2001–2006	180	4176.1	4.31	1	
2007–2012	92	2899.0	3.17	0.53 (0.36–0.79)	0.002
2013–2018	25	1405.4	1.78	0.19 (0.11–0.37)	<0.0001
**Class C**					
2001–2006	229	4176.1	5.48	1	
2007–2012	83	2899.0	2.86	0.47 (0.32–0.69)	<0.0001
2013-2018	42	1405.4	2.99	0.31 (0.18–0.53)	<0.0001

* *RR, rate ratio; CI, confidence interval*.

a *Adjusted for age and repeated events in the same patients*.

**Table 5:** Class B and C event rates among the study periods adjusted for age.

**Factors**	**Events (*n*)**	**Person-years**	Univariate IRR^*^ (95% CI^*^)	***P***	Multivariate IRR^*^ (95% CI^^*^^)	***P***
**CLASS B**						
**Period**						
2001–2006	180	4176.1	1		1	
2007–2012	92	2899.0	0.53 (0.36–0.79)	0.002	0.53 (0.36–0.79)	0.002
2013–2018	25	1405.4	0.20 (0.11–0.37)	<0.0001	0.19 (0.10–0.35)	<0.0001
**Follow-up**						
From	81	3365.4	1			
After birth	216	5115.0	1.78 (1.23–2.57)	0.002	1.86 (1.29–2.68)	0.001
**Date of birth**						
After 1996	908	4611.8	1			
Before 1996	89	3868.6	0.94 (0.65–1.36)	0.746		
**CLASS C**						
**Period**						
2001–2006	229	4176.1	1		1	
2007–2012	83	2899.0	0.47 (0.32–0.69)	<0.0001	0.47 (0.32–0.68)	<0.0001
2013–2018	42	1405.4	0.31 (0.18–0.53)	<0.0001	0.29 (0.17–0.49)	<0.0001
**Follow-up**						
From	74	3365.4	1			
After birth	280	5115.0	2.44 (1.66–3.60)	<0.0001	2.54 (1.74–3.71)	<0.0001
**Date of birth**						
After 1996	225	4611.8	1			
Before 1996	129	3868.6	1.19 (0.87–1.61)	0.275		

* *CI, confidence interval*.

Rates for specific events by calendar period are reported in Table 6. During the first calendar period, OIs (7.09/100 person-years) were the most common events: among them, severe bacterial infections (3.54/100 person-years), bacterial pneumonia (1.63/100 person-years) and polydermatomeric herpes zoster (0.45/100 person-years) were the most frequent. Other commonly reported events were haematological abnormalities (2.16/100 person-years). During the intermediate (2007–2012) and the last (2013–2018) calendar periods, many clinical event rates decreased. However, OIs, even if significantly reduced over time, remained the most frequent events still in 2013–2018, occurring at the rate of 6.76/100 person-years. Among OIs, the most common ones during the last calendar period were severe bacterial infections (0.71/100 person-years), *P. jirovecii* pneumonia (0.57/100 person-years) and cytomegalovirus disease (0.57/100 person-years) (Table 6). The proportion of *P. jirovecii* pneumonia and CMV disease were not decreasing during the three study periods, while all the others OIs drastically decreased overtime. Analysing data about these two opportunistic infections, we found that in the 70% of children *P. jirovecii* pneumonia and HIV infections had the same date of diagnosis, as well as in 60% of children with cytomegalovirus disease. The majority of children presented detectable VL when OIs occurred: 210 children out of 296 (70.9%) during the first calendar period, 52 children out of 97 (53.6%) during the second calendar period and 62 children out of 95 (65.3%) during the third calendar period. However, OIs occurred also in a minority of children with undetectable viremia [25 out of 296 (8.4%) in the first calendar period, 19 out of 97 (19.6%) in the second calendar period and 12 out of 95 (12.6%) in the third calendar period].

**Table 6:** Rates of the specific clinical events by calendar period and rate ratio (adjusted for age and repeated events in the same patient).

**Event class and calendar period**	**Events *(n)***	**Person-years**	**Rate ratio (events/100 person-years)**	Adjusted^a^ RR^*^ (95% CI^*^)	***P***
**Wasting syndrome**					
2001–2006	10	4176.1	0.31	1	
2007–2012	5	2899.0	0.17	0.57 (0.17–1.93)	0.396
2013–2018	0	1405.4	0.0		<0.0001
**Malignancies**					
2001–2006	2	4176.1	0.07	1	
2007–2012	4	2899.0	0.17	3.31 (0.54–20.25)	0.195
2013–2018	1	1405.4	0.07	1.03 (0.09–12.36)	0.978
**Encephalopathies**					
2001–2006	15	4176.1	0.36	1	
2007–2012	7	2899.0	0.24	0.79 (0.28–2.25)	0.659
2013–2018	8	1405.4	0.57	1.25 (0.47–3.33)	0.661
**Overall opportunistic infections**					
2001–2006	235	4176.1	7.09	1	
2007–2012	83	2899.0	3.35	0.46 (0.32–0.65)	<0.0001
2013–2018	36	1405.4	6.76	0.25 (0.14–0.46)	<0.0001
**Lymphoid interstitial pneumonia**					
2001–2006	6	4176.1	0.14	1	
2007–2012	5	2899.0	0.17	0.93 (0.25–3.48)	0.914
2013–2018	0	1405.4	0.0		<0.0001
**Hepatitis**					
2001–2006	18	4176.1	0.43	1	
2007–2012	9	2899.0	0.31	0.44 (0.15–1.28)	0.132
2013–2018	1	1405.4	0.07	0.004 (0.0005–0.03)	<0.0001
**Cardiopathy**					
2001–2006	2	4176.1	0.05	1	
2007–2012	1	2899.0	0.03	0.81 (0.07–9.36)	0.869
2013–2018	0	1405.4	0.0		<0.0001
**Nephropathy**					
2001–2006	2	4176.1	0.05	1	
2007–2012	4	2899.0	0.14	2.65 (0.35–20.00)	0.344
2013–2018	0	1405.4	0.0		<0.0001
**Anaemia/thrombocytopenia/neutropenia**					
2001–2006	90	4176.1	2.16	1	
2007–2012	52	2899.0	1.79	0.44 (0.22–0.89)	0.022
2013–2018	24	1405.4	1.71	0.28 (0.11–0.71)	0.007
**Severe bacterial infections**					
2001–2006	148	4176.1	3.54	1	
2007–2012	38	2899.0	1.31	0.33 (0.21–0.55)	<0.0001
2013–2018	10	1405.4	0.71	0.13 (0.06–0.28)	<0.0001
**Bacterial pneumonia**					
2001–2006	68	4176.1	1.63	1	
2007–2012	15	2899.0	0.52	0.26 (0.12–0.55)	<0.0001
2013–2018	0	1405.4	0.0		<0.0001
***P. jirovecii*** **pneumonia**					
2001–2006	17	4176.1	0.41	1	
2007–2012	5	2899.0	0.17	0.32 (0.06–1.69)	0.180
2013–2018	8	1405.4	0.57	1.90 (0.67–5.39)	0.229
**Cytomegalovirus disease**					
2001–2006	8	4176.1	0.19	1	
2007–2012	7	2899.0	0.24	0.65 (0.20–2.09)	0.469
2013–2018	8	1405.4	0.57	3.83 (1.30–11.30)	0.015
**Tracheobronchial/oesophageal candidiasis**					
2001–2006	16	4176.1	0.38	1	
2007–2012	5	2899.0	0.17	0.46 (0.14–1.56)	0.214
2013–2018	3	1405.4	0.21	0.14 (0.04–0.54)	0.004
**Disseminated herpes simplex infection**					
2001–2006	0	4176.1	0.0	1	
2007–2012	1	2899.0	0.03		<0.0001
2013–2018	2	1405.4	0.14		<0.0001
**Polydermatomeric herpes zoster**					
2001–2006	19	4176.1	0.45	1	
2007–2012	12	2899.0	0.41	0.86 (0.37–1.98)	0.719
2013–2018	0	1405.4	0.0		<0.0001
***Cryptosporidium spp*****. Infection**					
2001–2006	8	4176.1	0.19		
2007–2012	0	2899.0	0.0		
2013–2018	0	1405.4	0.0		
**Disseminated Mycobacterium infection**					
2001–2006	3	4176.1	0.07	1	
2007–2012	1	2899.0	0.03	0.37 (0.04–3.54)	0.387
2013–2018	2	1405.4	0.14	0.64 (0.07–6.11)	0.695

* *RR, rate ratio; CI, confidence interval*.

a *Adjusted for age and repeated events in the same patients*.

Significantly reduced rates of OIs were observed in children belonging to the more recent calendar periods and with high CD4^+^ T-lymphocyte percentage (>15%). At the same time, IRR was higher in children not followed up from birth, from abroad and with detectable viral loads (Table 7).

**Table 7:** Opportunistic infections rates among the study periods, adjusted for sex, starting of follow up, child's origin, use of cART, viral load, and CD4^+^%.

**Factors**	**Events (*n*)**	**Person-years**	Univariate IRR^*^(95% CI^*^)	***P***	Multivariate IRR^*^ (95% CI^*^)	***P***
**Period**						
2001–2006	235	4539.4	1		1	
2007–2012	83	2981.7	0.46 (0.32–0.65)	<0.0001	0.42 (0.29–0.63)	<0.0001
2013–2018	36	1462.4	0.25 (0.14–0.46)	<0.0001	0.21 (0.11–0.39)	<0.0001
**Sex**						
Male	169	4276.7	1			
Female	185	4706.8	0.97 (0.72–1.32)	0.861		
**Follow-up**						
From	79	3473.6	1		1	
After birth	275	5509.9	2.01 (1.39–2.90)	<0.0001	1.79 (1.25–2.56)	0.002
**Place of birth**						
Italy	264	7397.9	1		1	
Abroad	90	1585.6	1.44 (1.01–2.05)	0.043	1.39 (1.00–1.95)	0.048
**cART**						
No	139	2885.9	1			
Yes	215	6097.7	1.09 (0.82–1.46)	0.540		
**Viral load**						
Undetectable	35	2809.9	1		1	
Detectable	134	2164.3	4.30 (2.91–6.36)	<0.0001	2.80(1.88–4.20)	<0.0001
Missing	185	4009.4	2.62 (1.81–3.81)	<0.0001	1.77 (1.09–2.88)	0.021
**CD4**^**+**^ **T-lymphocyte percentage at the last cheque**						
≤ 15%	46	422.5	1		1	
>15%	108	4324.6	0.24 (0.16–0.35)	<0.0001	0.40 (0.27–0.59)	<0.0001
Missing	200	4236.4	0.37 (0.26–0.54)	<0.0001	0.82 (0.50–1.35)	0.445

*(Poisson regression model adjusted for age and repeated events in the same subject)*.

* *IRR, incidence rate ratio; CI, confidence interval*.

Seven cancers have been reported from 2001 to 2018: two in the first calendar period, four in the intermediate period and only one in the last calendar period. During the first calendar period cancers occurred at the rate of 0.07/100 person-years, at the rate of 0.17/100 person-years during the intermediate period (adjusted RR 3.31; CI: 0.54–20.25) and at the rate of 0.07/100 person-years during the last calendar period (adjusted RR 1.03; CI: 0.09–12.36) (Table 8). No significant reduction in the rates of cancers was observed in relation to sex, starting of follow up, child's origin, use of cART, VL, and CD4^+^ T-lymphocyte percentage (Table 9).

**Table 8:** Characteristics of malignancies occurred in the study population from 2001 to 2018.

	**Sex**	**Child's origin/Mother's origin**	**Age at first observation**	**First observation**	**Follow up**	**Clinical and immunological classes**	**Status (year of death)**	**Type of cancer**	**Year of the event**	**Age at the event [years]**	**Therapy at the occurrence of cancer (starting date)**	**Time on treatment before cancer**	VL^*^ at last cheque [Detect./Undetect.] (time between last cheque and cancer)	**CD4^**+**^ T-cell percentage at last cheque (time between last cheque and cancer)**
1	M	Italy/ Italy	0 m	1988	From birth	C 2	Adult care	Non-Hodg. B-cell lymph.	2003	14	DDI+ 3TC+ LPV+ RTV (2003)	13 y	Detect., (0 m)	31% (12 m)
2	M	Italy/ Italy	6 m	1994	After birth	C 3	Adult care	Burkitt lymph.	2007	14	FTC+ TDF+ SQV+ RTV+ ATV+ T20 (2007)	13 y	Undet., (1 m)	7% (13 m)
3	M	Italy/taly	0 m	1994	From birth	C 2	Dead (2009)	Burkitt lymph.	2009	15	FTC+ TDF+ LPV+ RTV (2007)	14 y	Undet., (0 m)	35% (1 m)
4	F	Italy/ Italy	0 m	2000	From birth	C 3	Adult care	Kaposi sarc.	2001	1	DDI + D4T (2000)	11 m	Detect., (2 m)	2% (1 m)
5	F	Italy/ Italy	6 y	2002	After birth	A 2	Dead (2012)	Non-Hodg. B-cell lymph.	2012	15	DDI+ D4T+ LPV+ RTV (2007)	9 y	Detect., (2 y)	31% (2 y 1 m)
6	F	Italy/ Italy	13 y 5 m	2010	After birth	C 3	Dead (2012)	Kaposi sarc.	2010	13	DDI+ 3TC+ ATV (2010)	<1 m	Detect., (0 m)	5.6% (0 m)
7	F	Italy/ Italy	15 m	2010	After birth	A 1	On follow up	Hodg. lymph.	2015	6	ABC+ 3TC+ ATV (2014)	3 y 8 m	Detect., (3 y)	27% (3 y)

* *Hodg, Hodgkin; lymph, lymphoma; sarc, sarcoma; VL, viral load; detect., detectable; undet., undetectable; y, years; m, months; DDI, didanosine; 3TC, lamivudine; LPV, lopinavir; RTV, ritonavir; FTC, emtricitabine; TDF, tenofovir disoproxil; SQV, saquinavir; ATV, atazanavir; T20, enfuvirtide; D4T stavudine; ABC, abacavir*.

**Table 9:** Malignancies rates among the study periods, adjusted for sex, starting of follow up, child's origin, use of HAART, viral load and CD4^+^ T-lymphocyte percentage (Poisson regression model).

**Factors**	**Events (*n*)**	Univariate IRR^*^ (CI)	***P***	Multivariate IRR^*^ (CI^*^)	***P***
**Period**					
2001–2006	2	1			
2007–2012	4	3.31 (0.54–20.25)	0.195		
2013–2018	1	1.03 (0.09–12.36)	0.978		
**Sex**					
Male	3	1			
Female	4	0.83 (0.18–3.81)	0.807		
**Follow up**					
From birth	3	1			
After birth	4	0.94 (0.20–4.47)	0.937		
**Child's origin**					
Italy	7	1			
Abroad	0	1.26 (0.79–2.02)	0.334		
**HAART**					
No	0	1			
Yes	7	1.38 (0.88–2.14)	0.158		
**Viral load**					
Undetectable	3	1			
Detectable	2	0.52 (0.08–3.28)	0.485		
Missing	2	0.32 (0.05–2.08)	0.235		
**CD4**^**+**^ **T-lymphocyte percentage at the last cheque**					
≤ 15%	3	1			
>15%	2	0.07 (0.01–0.44)	0.004		
Missing	2	0.06 (0.01–0.38)	0.003		
**CD4**^**+**^ **T-lymphocyte percentage at the last cheque**					
≤ 25%	3	1			
>25%	2	0.28 (0.04–1.74)	0.171		
Missing	2	0.19 (0.03–1.24)	0.083		

* *IRR, incidence rate ratio; CI, confidence interval*.

Overall, non-Hodgkin B-cell lymphoma (*n* = 2), Burkitt lymphoma (*n* = 2), Kaposi sarcoma (*n* = 2), and Hodgkin lymphoma (*n* = 1) were observed. Five out of seven cancers (71.4%) occurred in children who had detectable VL at the last cheque performed before cancer occurrence. At the same time, four children out of seven (57.1%) presented high CD4^+^ T-cell counts (>25%) at the last cheque. Moreover, five children with cancer out of seven (71.4%) were on cART for more than 5 years before the occurrence of the tumour (Table 8). HIV-1 encephalopathy was observed in 25 children: 15 in the first study period, 7 in the second one and 8 in the third one. Incidence rates of HIV-1 encephalopathy were: 0.36/100 person-years during the first study period, 0.24/100 person-years during the second one and 0.57/100 person-years during the third one, with no significant reduction overtime (Table 6). All the cases were newly diagnosed in each period and never recorded previously. Most cases of encephalopathy (20 out of 25, i.e., 80%) occurred in children who were not followed up from birth. Moreover, we observed that six children aged from 0 to 4 years old reported psychomotor delay (non-meeting CDC diagnostic criteria for HIV-1 encephalopathy): acquired motor deficits, failure to attain or loss of psychomotor developmental milestones, neurocognitive delay.

## Discussion

**Figure 3:**
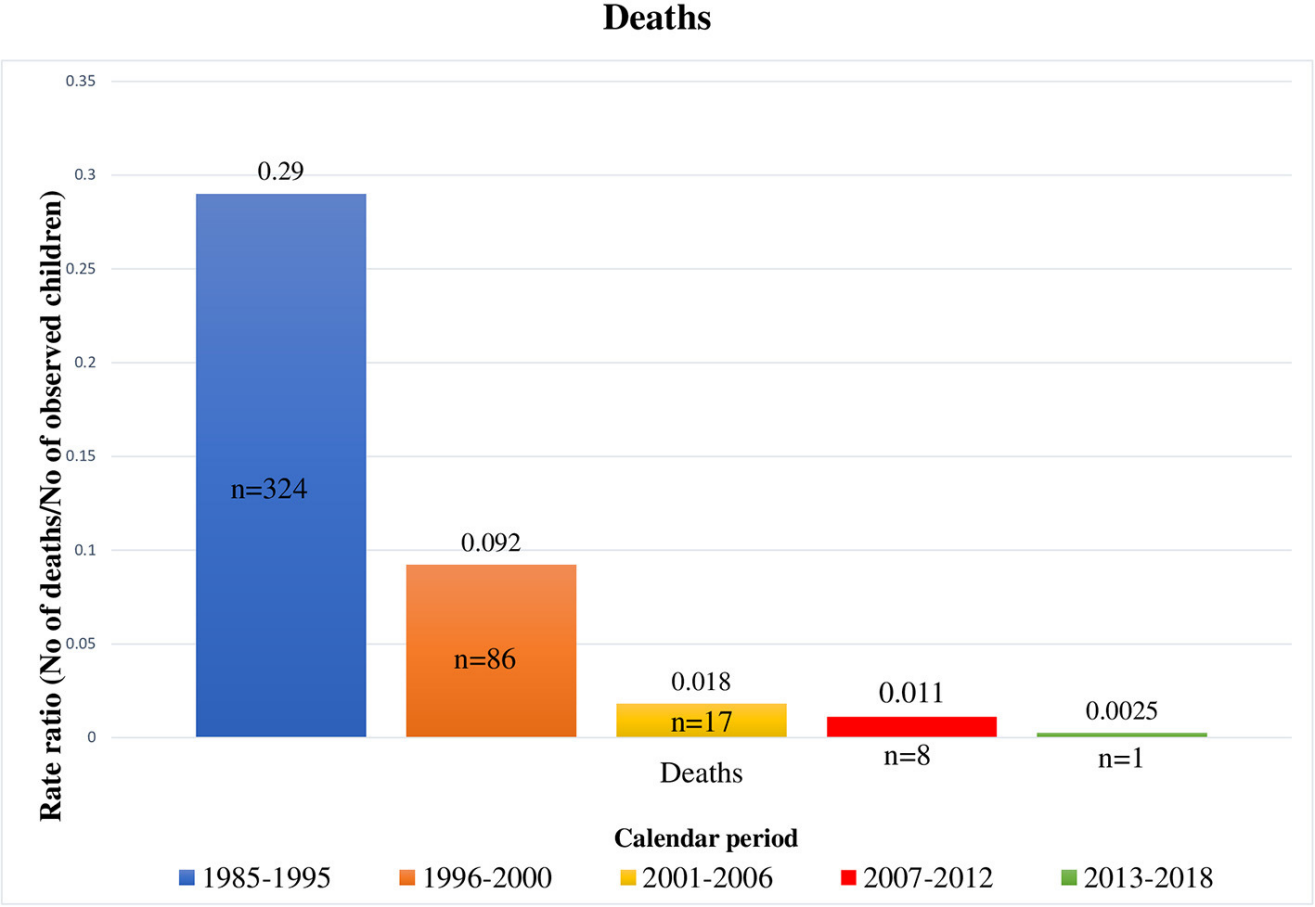
Deaths occurred in HIV-1 infected children by calendar period, from 1985 to 2018 [1985–1995 and 1996–2000 periods refer to our previous results (18)].

This study described changes in mortality rates, causes of death, and clinical events during the most recent years of the cART era (from 2001 to 2018). Mortality rates significantly decreased over time, not only during our study calendar periods but also in comparison to data reported in our previous study (19) (Figure 3).

Mortality risk in the cART era is far lower than in the pre-cART era, as previously reported (23–25). However, even though life expectation of perinatally HIV-1 infected children has lengthened and mortality rates in Western European countries such as Netherlands and Spain (26, 27) are decreasing (0.3 per 100 person-years from 1996 to 2012 and 0.0 in 2009, respectively), deaths have not yet disappeared.

It is already well-known that early diagnosis, strict follow up from birth, early, and effective therapy is fundamental to keep the disease under control and to prevent death and progression toward AIDS (11, 12). Indeed, significantly reduced rates of death were observed in children with high CD4^+^ T-lymphocyte percentage (>15%). At the same time, almost all the dead children (88.5%) presented detectable VL at the last cheque before death, and more than half had low CD4^+^ T-cell counts (<15%) at last cheque, confirming current data in literature (15, 16, 28–30). Prior to the accessibility to cART in Europe, almost 20% of infants experienced early disease progression in the first year of life and 10% died (45, 46). The South African controlled randomised trial and the European collaborative cohort study demonstrated that early use of cART (before 5 or 3 months of life, respectively) slows the progression of the disease (12). Indeed, AIDS events precociously occurred (before the age of 2 years) in children treated after 3 months, while the rate of occurrence of AIDS or death of infants belatedly treated was similar to historical reports of HIV infected infants not treated with cART (47, 48). Early initiation of cART was also associated with a significantly reduced risk of HIV encephalopathy (49) as well as with limited immunological progression (14). In line with these results, we noticed that mortality risk in children not in follow up from birth was higher (even if not significantly) than in children followed up from birth and, therefore, early treated with cART. However, no difference in mortality risk was found between patients born before and after 1996 (year of cART introduction). Analysing more in detail the characteristics of dead children, the mortality data are not very reassuring if considering that more than 70% of dead children had initiated treatment when they were ≥6 months old, even if 24 out of the 26 dead children (92.3%) were born in Italy. On the one hand, this result confirms that children who are belatedly diagnosed and treated are at greater risk of death in percentage terms, on the other hand it highlights how even in a Western country like Italy many children still escape an early diagnosis and treatment from birth.

It must also be highlighted that more than one-fifth of the dead children had received cART for less than a month and almost the same amount for <10 years in any case. Unfortunately, we do not have enough information to recognise the causes of these failures (i.e., poor adherence, viral resistance).

**Figure 4:**
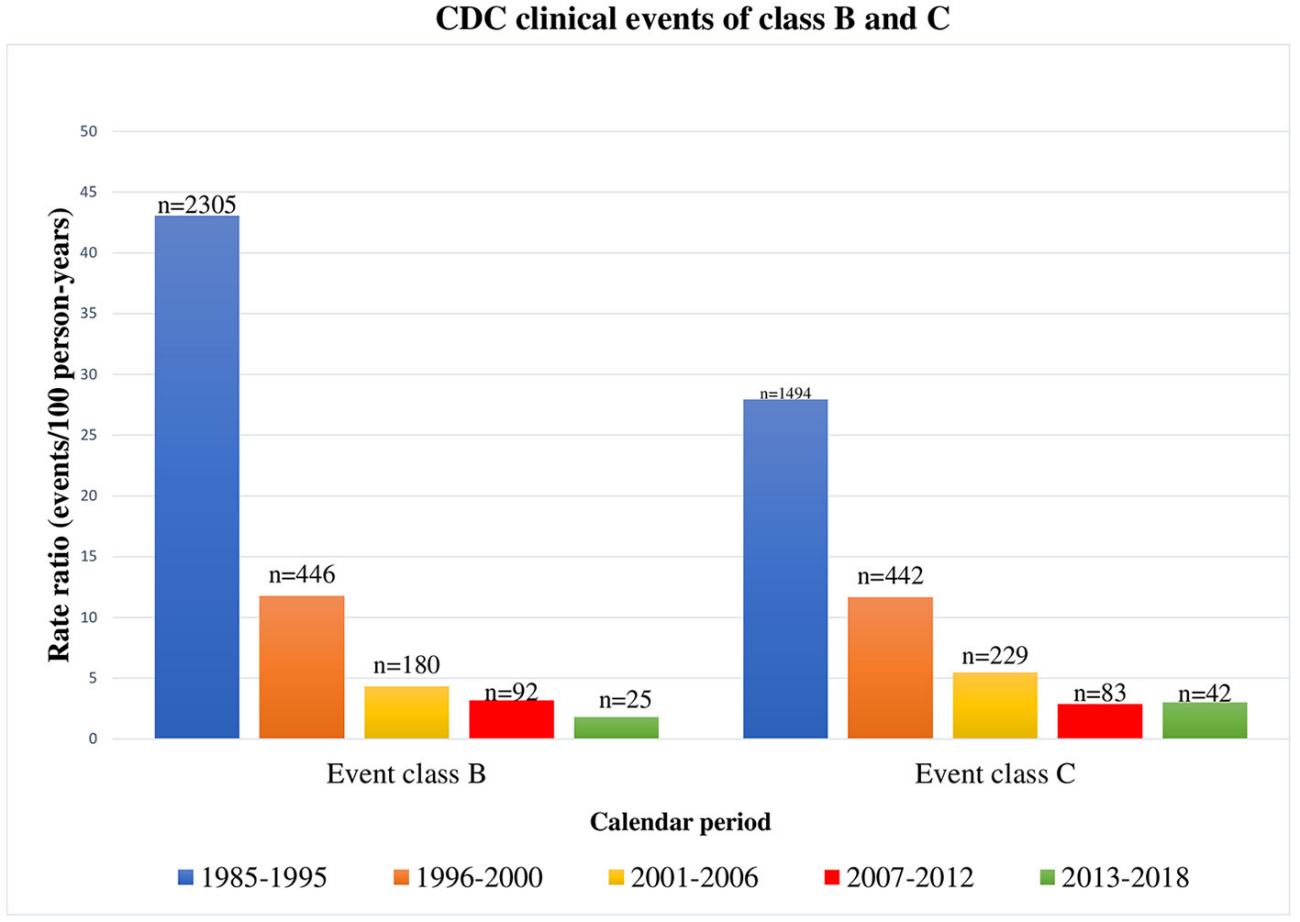
CDC clinical events of class B and C occurred in HIV-1 infected children by calendar period, from 1985 to 2018 [1985–1995 and 1996–2000 periods refer to our previous results (18)].

A deep decrease in class B and C clinical events occurred during the three study periods (from 2001 to 2018). Moreover, comparing clinical events occurred from 2001 to 2018 to the events occurred from 1985 to 2005 (19), a significant reduction in both class B and C events can be noticed (Figure 4). As already reported in literature (11, 12), class B and C event rates were significantly reduced in children followed up from birth, confirming the importance of early diagnosis and treatment to slow disease progression.

Cancer rates significantly decreased compared to those of the first two periods of the previous study (19), but remained almost stable if compared to the third calendar period of the same study (19) and during our three study periods. As reported in a recent case-control study of 1,307 perinatally HIV-1 infected Spanish children (31), and in the US HIV/AIDS Cancer Match Study (32), the overall rate of cancers did not significantly change in the last years, remaining high even in the cART era. However, in contrast with literature, which reported that AIDS related malignancies declined during the cART era while the incidence of non-AIDS related cancers increased over time (31, 33), in our study, the most common cancers remained Burkitt lymphoma, Kaposi sarcoma and non-Hodgkin B-cell lymphoma, as observed in low-income-resource countries (34, 35). No case of leiomyosarcoma was found, even if it is now the second most frequent malignancy in HIV-infected children in the USA (36). Any significant relation between the immunovirological status (expressed by CD4^+^ T-cell count and viral load) and cancer risk was found. Even if 71.4% of the children were on treatment for more than 5 years before cancer occurrence, almost three-quarters of the cancers occurred in children with detectable viral loads at the last cheque, suggesting, despite the long time spent in treatment, poor adherence to the therapy itself.

**Figure 5:**
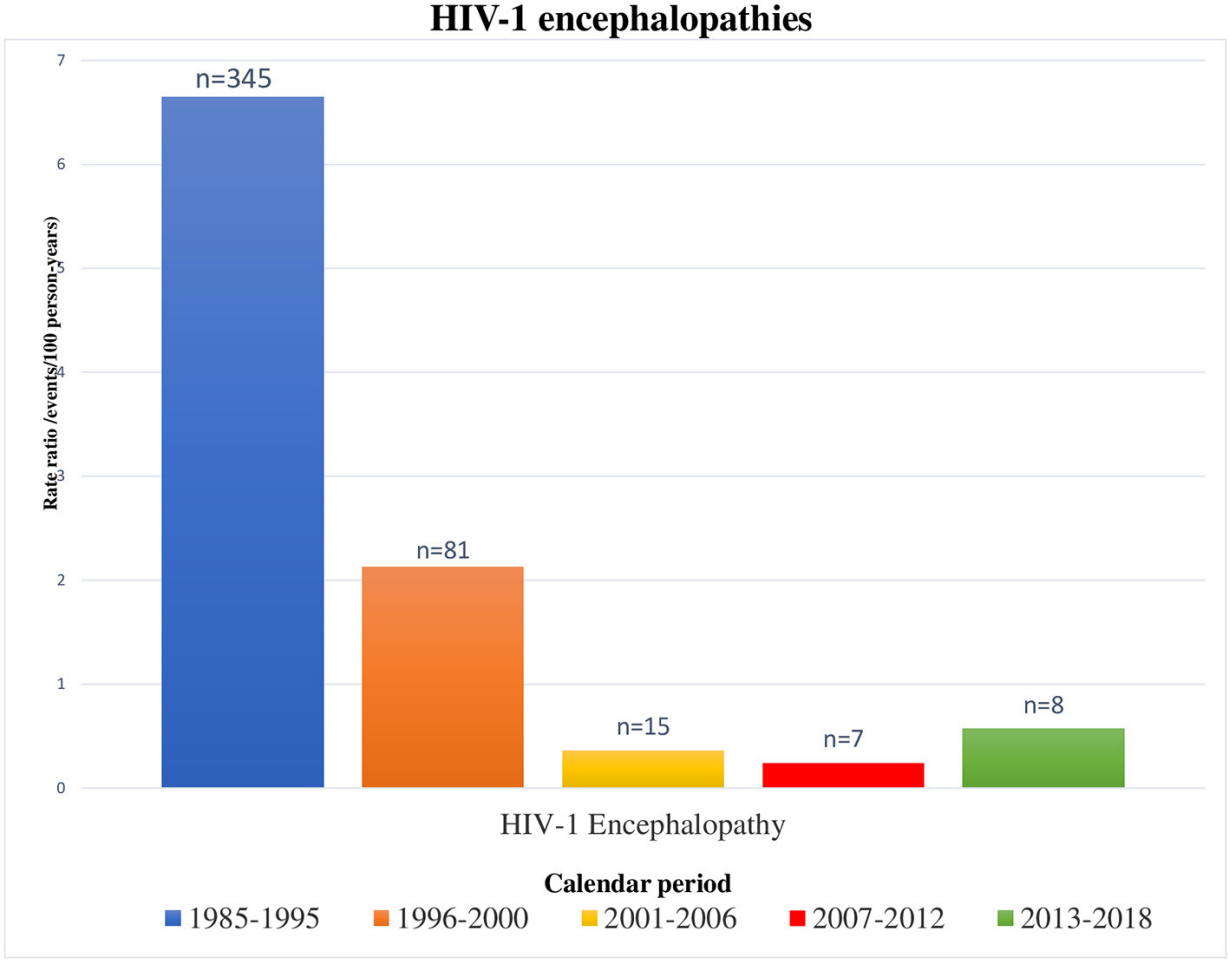
Encephalopathies/neurocognitive delay occurred in HIV-1 infected children by calendar period, from 1985 to 2018 [1985–1995 and 1996–2000 periods refer to our previous results (18)].

OIs rates significantly reduced during the cART era, in parallel to the restoration of the immune system activity (15, 17, 37, 38). Indeed, OIs more frequently occurred in children with detectable viral loads and low CD4^∧^+ T-lymphocyte counts (<25%). Overall OIs, HIV-1 hepatitis, lymphoid interstitial pneumonia, severe bacterial infections, bacterial pneumonia, disseminated herpes simplex infection, and polydermatomeric herpes zoster rates have been significantly reduced from 1985 to 2018 (*P* < 0.0001). On the other hand, *Pneumocystis jirovecii* pneumonia and CMV infection rates have not been significantly reduced over time. They are probably not decreasing (differently from other OIs) in relation to two factors: certainly, diagnostic techniques have improved thanks to implementation of PCR tests; moreover, these types of infection are often onset diagnosis of HIV infection, when viral loads are obviously still detectable. OIs also occurred in children with undetectable viremia. Therefore, it should be crucial to investigate the functional immunologic aspects, too. As already reported in the literature, B-cell and T-cell profiles alterations could correlate with the worse immune response to pathogens (39–42). In our study, HIV-1 encephalopathy was observed in 25 children. Despite a significant reduction (*P* < 0.0001) observed in our previous study (19) from 1985 to 2005 (probably related to the large-scale introduction of cART), there were no statistically significant reductions in HIV-1 encephalopathy incidence from 2001 to 2018 (Figure 5).

Eighty percent of encephalopathies occurred in children who were not followed up from birth. We also observed children who developed acquired motor deficits, failure to attain or loss of psychomotor developmental milestones, neurocognitive delay (non-meeting CDC diagnostic criteria for HIV-1 encephalopathy). This finding reflects what observed in other recent studies: even if the introduction of cART reduced the rates of HIV-1-related encephalopathy (43, 44, 50), milder neurocognitive deficits (lower total intelligence quotient, language impairment, poorer working memory, gross and fine motor functioning, visual-motor impairment) are diagnosed more and more frequently (51–56).

Our population study was conducted on a representative cohort of not-selected children, prospectively followed up. Therefore, this assessment reflects what is actually occurring in the paediatric population and could be helpful for experts who are taking care of perinatally HIV-1 infected children in Western countries. The study can be biassed in various ways: underreporting is possible, only basic data can be collected; children lost to follow-up were excluded from the study, therefore if they experienced clinical events during the study period, these data haven't been reported; data about HIV-infected people aged >18 years old are missing; diagnoses of clinical events have been collected in numerous different hospital centres implying a certain heterogeneity in the diagnostic criteria (in particular for clinical events as HIV-1 encephalopathies, whose diagnosis is purely clinical.) However, we believe that none of these potential biases significantly affected our results. *The Italian Register for HIV Infection in Children* is one of the largest cohorts of children perinatally infected with HIV-1 and highly representative of the entire population of Italian HIV-1-infected children (9). In conclusion, we provide evidence for a progressive reduction in mortality and morbidity in perinatally HIV-1 infected children during the cART era in a Western country. At the same time, we underline that the number of deaths has not completely reset, although the extensive use of cART and, despite the longer life expectancy for HIV-1 infected children during the cART era, severe bacterial infections and cancers continue to occur at higher rates than in general paediatric population. The majority of children who died or developed HIV-related clinical events presented detectable viral loads. This finding suggests that there is probably a serious problem with adherence to therapy and considerable efforts still need to be made in order to implement strategies to improve the adherence itself (57). Our data shows that we cannot yet consider HIV infection completely harmless. It must be underlined that only almost 30% of the dead children were followed from birth and that the majority of them had belatedly initiated cART (at age ≥6 months), confirming that late diagnosis and treatment correlates with higher mortality. Mortality risk and class B and C clinical events incidence were higher in children not followed up from birth. Therefore, it seems to be clear that many HIV related events probably wouldn't have occurred if diagnosis and therapy were more precocious and if adherence to the treatment was greater. As exposed in a recent study by Di Biagio et al. (58), numerous efforts, both medical and organisational, are still needed to improve the survival and quality of life of children with perinatal HIV-1 infection. For this purpose, it is essential to ensure early diagnoses, to start treatment early and to improve adherence to therapy in the paediatric population.

## Data Availability Statement

Publicly available datasets were analysed in this study. This data can be found here: Italian Register for HIV Infection in Children.

## Ethics Statement

The studies involving human participants were reviewed and approved by Ethics Committee University of Florence. Written informed consent to participate in this study was provided by the participants' legal guardian/next of kin.

## Author Contributions

EC and FL contributed to conception, design of the study, and wrote the first draught of the manuscript. CL performed the statistical analysis. All authors contributed to manuscript revision, read, and approved the submitted version.

## Italian Register for HIV Infection in Children

Elena Chiappini, Luisa Galli, Pier Angelo Tovo, Carlo Giaquinto, Catiuscia Lisi. Other collaborators who refer to the Italian Register for HIV Infection in Children and aided the efforts of the authors: Maurizio Ruggeri (Bergamo), Francesco Baldi, Giacomo Faldella (Bologna), Piergiorgio Chiriacò (Brindisi), Carlo Dessì (Cagliari), Maria Grazia Pantò (Catania), Elisa Anastasio (Catanzaro), Luisa Abbagnato (Como), Maria Rita Govoni (Ferrara), Maurizio Bigi (Forlì), Elisabetta Bondi (Genova), Riccardo Borea, Giovanni Cenderello (Imperia), Donato Tommasi (Lecce), Raffaella Pinzani (Milano) Ernesto Renato Dalle Nogare, Marcello Saitta (Palermo), Leonardo Felici (Pesaro), Rita Consolini (Pisa), Angelo Antonellini (Ravenna), Gianfranco Anzidei, Orazio Genovese, Salvatore Catania, Anna Maria Casadei (Roma), Paolina Olmeo (Sassari) Letizia Cristiano (Taranto), Vincenzo Portelli (Trapani), Marco Rabusin (Trieste), Annamaria Plebani (Varese).

## Conflict of Interest

The authors declare that the research was conducted in the absence of any commercial or financial relationships that could be construed as a potential conflict of interest.
